# Variation among Consent Forms for Clinical Whole Exome Sequencing

**DOI:** 10.1007/s10897-017-0127-2

**Published:** 2017-07-08

**Authors:** Sara A. Fowler, Carol J. Saunders, Mark A. Hoffman

**Affiliations:** 1 Department of Biomedical and Health Informatics, University of Missouri-Kansas City, Kansas City, MO USA; 2 Center for Pediatric Genomic Medicine, Children’s Mercy Hospital, Kansas City, MO USA; 3 Department of Pathology and Laboratory Medicine, Children’s Mercy Hospital, Kansas City, MO USA; 4 School of Medicine, University of Missouri-Kansas City, Kansas City, MO USA; 5 Children’s Mercy Hospital, 2401 Gilham Road, Kansas City, MO 64108 USA

**Keywords:** Informed consent, Exome sequencing, Bioethics, Informatics

## Abstract

The goal of this study was to explore variation among informed consent documents for clinical whole exome sequencing (WES) in order to identify the level of consistency with the recommendations from the American College of Medical Genetics and Genomics (ACMG) and the Presidential Commission for the Study of Bioethical Issues (Bioethics Commission) regarding informed consent for clinical WES. Recommendations were organized into a framework of key points for analysis. Content analysis was conducted on a sample of informed consent documents for clinical WES downloaded from 18 laboratory websites. We observed considerable variability in the content of informed consent documents among the sample of 18 laboratories. The mean Flesch-Kincaid Grade Level, a measure of readability, of the consent forms was 10.8, above the recommended 8th grade level. For each of the individual ACMG and Bioethics Commission recommendations, the frequency of inclusion ranged from 11% to 100%. For the overall list of 18 consent items, inclusion ranged from 11 to 17 items (Mean = 13.44, Mode = 14). This analysis will be useful to laboratories that wish to create informed consent documents that comply with these recommendations. The consistent use of standardized informed consent process could improve communication between clinicians and patients and increase understanding of genetic testing.

## Introduction

Whole Exome Sequencing (WES) has recently been extensively incorporated into clinical care to identify complex hereditary disorders that are difficult to diagnose because of unusual presentation or rare occurrence (Presidential Commission for the Study of Bioethical Issues (Bioethics Commission) 2012; Green et al. 2013; Tacik et al. 2015). Secondary findings (SFs) are test results that are not the primary object of study but that are sought because of potential health importance for a patient and their biological relatives. As the use of WES increases, ethical challenges about the appropriate management of SFs are a growing concern (ACMG 2013; Bioethics Commission 2013; Crawford et al. 2013; Hull and Berkman 2014; Roche and Berg 2015; Weiner 2014; Wolf 2015). Important ethical issues posed by SFs include the role of patients in choosing whether or not to receive these results, whether clinicians should seek SFs, whether SFs for adult-onset disorders should be disclosed to children, and obligations of patients and physicians to disclose SFs to family members of patients (Berg et al. 2015; Green et al. 2013; Gutmann 2013; Klitzman et al. 2013). Clinicians would benefit from consistent application of guidelines on returning SFs (Berg et al. 2015; Green et al. 2013; Gutmann 2013; Klitzman et al. 2013). The establishment of such standards would support clinicians’ professional judgment in specific clinical situations and assist genetic counselors in targeting information provided to patients, allowing for an efficient informed decision making process (Berg et al. 2015; Bioethics Commission 2013; Green et al. 2013).

Patients need to comprehend the benefits and risks of WES in order to prepare for results that may be emotionally, socially, or financially upsetting (McGuire and Gibbs 2006; Rigter et al. 2013; Simon et al. 2012). Trust between the clinician and patient could be compromised if there is not adequate disclosure (McGuire and Gibbs 2006). While context-specific differences allow for variance between consent models, the development of customary language for informed consent and return of SFs will improve communication between clinicians and patients (Bioethics Commission 2013; Weiner 2014). Standardized systems can facilitate health information exchange so that data can be more easily aggregated and studied, thereby permitting empirical study of patients’ choices following informed consent for clinical WES and evidence-based recommendations to patients during pre- and post-test genetic counseling (Berg et al. 2013; Bioethics Commission 2012; Green et al. 2013; McGuire and Gibbs 2006; Weiner 2014).

Many adults lack the necessary literacy skills to understand patient health care-related materials (Mueller et al. 2010). Multiple studies have demonstrated that patient education resources are too difficult for approximately one-quarter of US adults to read and comprehend (Badarudeen and Sabharwal 2010; Mueller et al., 2010; Paasche-Orlow et al. 2003). Because literacy difficulties are prevalent, many Institutional Review Boards recommend that language should be aimed at the 8th grade reading level (Badarudeen and Sabharwal 2010; Gargoum and O’Keeffe 2014; Simon et al. 2012). The quantity and complexity of information included in a consent form for WES makes it challenging to meet the 8th grade reading level target (Badarudeen and Sabharwal 2010; Gargoum and O’Keeffe 2014; Paasche-Orlow et al. 2003; Simon et al. 2012). Readability tools such as the Flesch-Kincaid Readability tests can be used to assess the length and structure of the text, but do not measure other factors that influence comprehension such as layout, illustrations, or motivation of the reader (Badarudeen and Sabharwal 2010; Paasche-Orlow et al. 2003).

Consenting a patient for clinical WES requires striking a balance between information overload and uninformed consent (Berg et al. 2013; Rigter et al. 2013). Attributes that may be significant to a patient’s informed decision to learn SFs include lifetime risk, treatability and gravity of the condition, and cost (Roche and Berg 2015). Procedures for providing informed consent vary in whether the patient “opts-in”, actively choosing to receive SFs; “opts-out”, actively choosing *not* to receive SFs; or, makes “stratified” choices of which genes will be analyzed and which types of SFs may be returned (e.g., medically actionable, carrier status, variants of unknown significance) (McGuire and Gibbs 2006).

There is conflicting guidance about whether to seek and how to manage SFs (Gutmann 2013). Most SFs have limited medical actionability, leading to a lack of consensus regarding their routine disclosure (Roche and Berg 2015). The ACMG recommends required reporting of likely pathogenic or pathogenic variants found in 56 genes, referred to as the “minimum list”, regardless of the indication for which the clinical sequencing was ordered (Green et al. 2013). In contrast, guidance from the Bioethics Commission finds no ethical duty for clinicians to search for genetic results that are not relevant to the clinical indication for sequencing, instead recommending that practitioners and policy makers deliberate to identify specific criteria for determining when it is ethically permissible or obligatory to disclose SFs (Weiner 2014).

### Ethical Considerations for Secondary Findings

The standards for clinical genomic sequencing recognize a distinction between providing SFs to adults versus children and adolescents (Green et al. 2013). Scientific advances during a child’s lifetime can compound the current unknown risks raised by genome-scale sequencing (Bioethics Commission 2012). Sequencing data obtained from a minor could be widely shared before they reach an age to self-determine data sharing limits, diminishing their autonomy as adults (Bioethics Commission 2012). Clinical practice guidelines generally recommend that only information that is clearly actionable in childhood be disclosed and that the decision to learn about adult-onset conditions be delayed until the age of majority out of respect for the child’s development (Hull and Berkman 2014; Roche and Berg 2015). However, the ACMG recommends that seeking and reporting SFs for variants with a high likelihood of causing disease not be limited by the age of the person being sequenced (Green et al. 2013).

Information about genetic variants has health implications for the patient’s family since first-degree biological relatives share 50% of their genetic information (Bioethics Commission 2013; Egalite et al. 2014; Wolf 2015). Genetic testing targeting specific genes in an affected patient may reveal that a patient’s relative has, is at risk for, or is a carrier of a specific disease (Bioethics Commission 2013; Egalite et al. 2014; Wolf 2015). In this situation, the clinician may be confronted with conflicting duties to protect the patient’s privacy and to warn biological relatives of shared risk (Ross et al. 2013; Simon et al. 2012; Wolf 2015).

### Recommendations of the ACMG

In 2013, the ACMG Board of Directors established a Working Group to evaluate “the utility of making recommendations for analyzing and reporting incidental findings from sequencing in the clinical context” (Green et al. 2013, p. 4). The resulting consensus guidelines are an instructive resource for health care providers who deliver medical genetic services (Green et al. 2013; Hull and Berkman 2014; Weiner 2014). The ACMG also recognizes the “right *not* to know”, to opt-out of the analysis of SFs (ACMG 2015; Egalite et al. 2014; Hull and Berkman 2014; Weiner 2014). Regarding the informed consent process, the ACMG recommends addressing issues including interpretive uncertainty, privacy, and possible impact on family members (ACMG 2013; Reinke 2015).

### Recommendations of the Bioethics Commission

The Bioethics Commission recommended the creation of evidence-based practice guidelines on return of genomic sequencing results, recognizing that a robust informed consent process is necessary for ethical clinical care (Bioethics Commission 2012, 2013; Weiner 2014). The informed consent process should effectively apprise individuals without undermining their ability to make voluntary choices (Bioethics Commission 2012).

### Study Objectives

The guidelines of the ACMG and Bioethics Commission offer discrete criteria that can contribute to a framework for reporting SFs, standardizing the informed consent process, improving communication between clinicians and patients, and increasing understanding of genetic testing. We evaluated the use of these criteria in publically available patient consent forms among diagnostic laboratories offering WES. We also evaluated the variability of the forms using standard comprehensibility analyses.

## Materials and Methods

### Data Collection

We collected a convenience sample of informed consent documents for clinical WES. We identified laboratories that conduct clinical WES from the National Center for Biotechnology Information’s (NCBI) Genetic Testing Registry (www.ncbi.nlm.nih.gov/gtr/), GeneTests (www.genetests.org/), and NextGxDx (www.nextgxdx.com/). Each of these sites was searched for Clinical Laboratory Improvement Amendment (CLIA)-certified laboratories in the United States that claim to provide clinical WES.

### Document Description and Unit Definition

We found a range of documents and addenda for provision of informed consent, including: ‘Patient Consent Form’, ‘Consent Form, Proband Only’, ‘Consent Form, Family Trio’, ‘Test Requisition Form’, ‘Expanded Secondary Findings Request Form’, ‘Raw Sequence Data Consent Form’, and ‘Authorization for Participation in a Research Protocol’. Given the variation in forms, the combination of documents from each laboratory was organized to comprise a consent form unit of analysis (CFU) as they would be presented to a patient (i.e. primary consent template with addenda).

### Procedures and Data Analysis

A content analysis matrix was created by analyzing the recommendations from two ACMG policy statements, *ACMG Recommendations for Reporting of Incidental Findings in Clinical Exome and Genome Sequencing* (Green et al. 2013), and *Points to Consider for Informed Consent for Genome/Exome Sequencing* (ACMG 2013)*,* and two Bioethics Commission reports, *Privacy and Progress in Whole Genome Sequencing* (Bioethics Commission 2012), and *Anticipate and Communicate: Ethical Management of Incidental and Secondary Findings in the Clinical, Research, and Direct-to-Consumer Contexts* (Bioethics Commission 2013). Key points recommended for inclusion were identified and organized by common themes.

As CFU documents were collected, consent items not directly associated with the ACMG and Bioethics Commission recommendations were apparent and prompted the creation of a secondary list of additional content features. These additional items included consent for return of raw sequence data, authorization for transfer of information to another health care provider, and items associated with legal concern, including the OHRP recommendation to explain the Genetic Information Nondiscrimination Act (GINA), (2008) (U.S. Department of Health and Human Services, Office for Human Research Protections (OHRP) 2009), the New York Civil Rights statute for sample storage, and nondisclosure of proprietary data (New York Department of Health 2011). These five additional content items were included in the coding framework separately in order to distinguish analysis of ACMG and Bioethics Commission guidance from other legal or practical information that may be useful to clinicians (Table 1).

**Table 1:** Content analysis coding matrix

ACMG & bioethics commission recommendations	
Key point	Description
1. Description of WES	Briefly describe WES and analysis
2. Purpose for WES	State how the data will be used
3. Benefits and risks of WES*	Define potential benefits and risks of the procedure
4. Uncertainty of results*	Explain the limitations of testing
5. Follow-up if results are updated	Describe laboratory policy regarding re-contact of referring physician and/or patient as new knowledge is gained about significance of particular results
6. Results returned to whom	State to whom the findings will be communicated
7. Describe results returned to proband*	Explain the scope of data and information that might be returned to the individual
8. Results excluded from report*	Explain types of results that will not be returned
9. Define SFs*	Define the secondary findings that are possible, or likely, to arise or be sought from the procedure
10. Options for ACMG minimum list results*	Describe the laboratory policy for disclosing the ACMG minimum list
11. Return SFs for minors*	Describe steps to be taken upon discovery of secondary findings for minors
12. Disclose SFs to relatives*	Explain steps to be taken upon discovery of secondary findings with potential implications for family members
13. Disclose carrier status for recessive disorders	Explain options for receiving information derived indicating carrier status for recessive disorders
14. Sample may be shared in databases*	Request permission to provide individually identifiable results to databases
15. Request to use sample for research	Request permission to use the data for research purposes
16. Who has access to sequence data	Describe who has access to the data generated in the course of clinical WES
17. Opportunity for genetic counseling	Describe the options for genetic counseling
18. Risk discovering misattributed parentage	Explain laboratory policy for providing information indicating misattributed parentage
Additional Recommendations	
Key Point	Description
1. Risk of insurance discrimination/GINA	Describe the protections provided by the Genetic Information Nondiscrimination Act (GINA)
2. Transfer results to another clinician	Laboratory provides option for transfer of results to another clinician
3. Return raw data file	Laboratory provides option for return of raw data file to referring physician or another clinician
4. Samples from NY destroyed in 60 days	Explain that samples from NY must be destroyed within 60 days of testing unless patient consents to retention
5. Some genetic information is proprietary	Include disclaimer that some genetic information may be proprietary and the laboratory may not be able to analyze or report certain results

*Recommended by both ACMG and Bioethics Commission

Each CFU was reviewed using the coding matrix. A hard copy of the CFU was read by a single reviewer checking for direct and implicit language addressing each recommendation on the coding matrix. When wording associated with a recommendation was identified it was highlighted and coded. This review was repeated to confirm that the matrix was applied consistently and accurately. The identified text for each code was entered into an Excel spreadsheet and qualitatively assessed for continuity with the recommendation. The text data was coded as present (1) or missing (0) in another spreadsheet for quantitative analysis. The quantitative data was checked for missing items. If a recommendation was missing, the CFU was read a third time to confirm that the recommendation was not present.

Descriptive statistics were used to summarize the sample and Fisher’s Exact tests compared differences between laboratory types.

The readability of each CFU was measured using the Flesch-Kincaid Grade Level score, a measure of the number of syllables per word and words per sentence, corresponding to the number of years of education required to comprehend the text (Gargoum and O’Keeffe 2014; Kincaid et al. 1975; Simon et al. 2012). The grade level score of each CFU was calculated automatically using Microsoft Word 2010.

## Results

Between January and February 2016, we identified 25 CLIA-certified laboratories registered with GeneTests, GTR and NextGxDx (now Concert Genetics) as providing clinical WES. GeneTests currently provides information about 708 laboratories; GTR, 491. Concert Genetics does not identify the number of labs in their database. The website for each of these 25 laboratories was accessed to confirm that it was CLIA-certified and provided clinical WES, as listed on the registry. Two of these laboratories were excluded because they provided WES only for research purposes. The remaining 23 laboratory websites were searched for links to their informed consent documents; five did not post their informed consent documents for public download. We obtained informed consent documents from 18 of the remaining 23 (78%) organizations.

Our final sample of 18 laboratories utilized 29 different informed consent forms and addenda. The CFUs were categorized by institution type as commercial laboratories (*n* = 8), or academic laboratories affiliated with a hospital or university (*n* = 10).

**Fig. 1:**
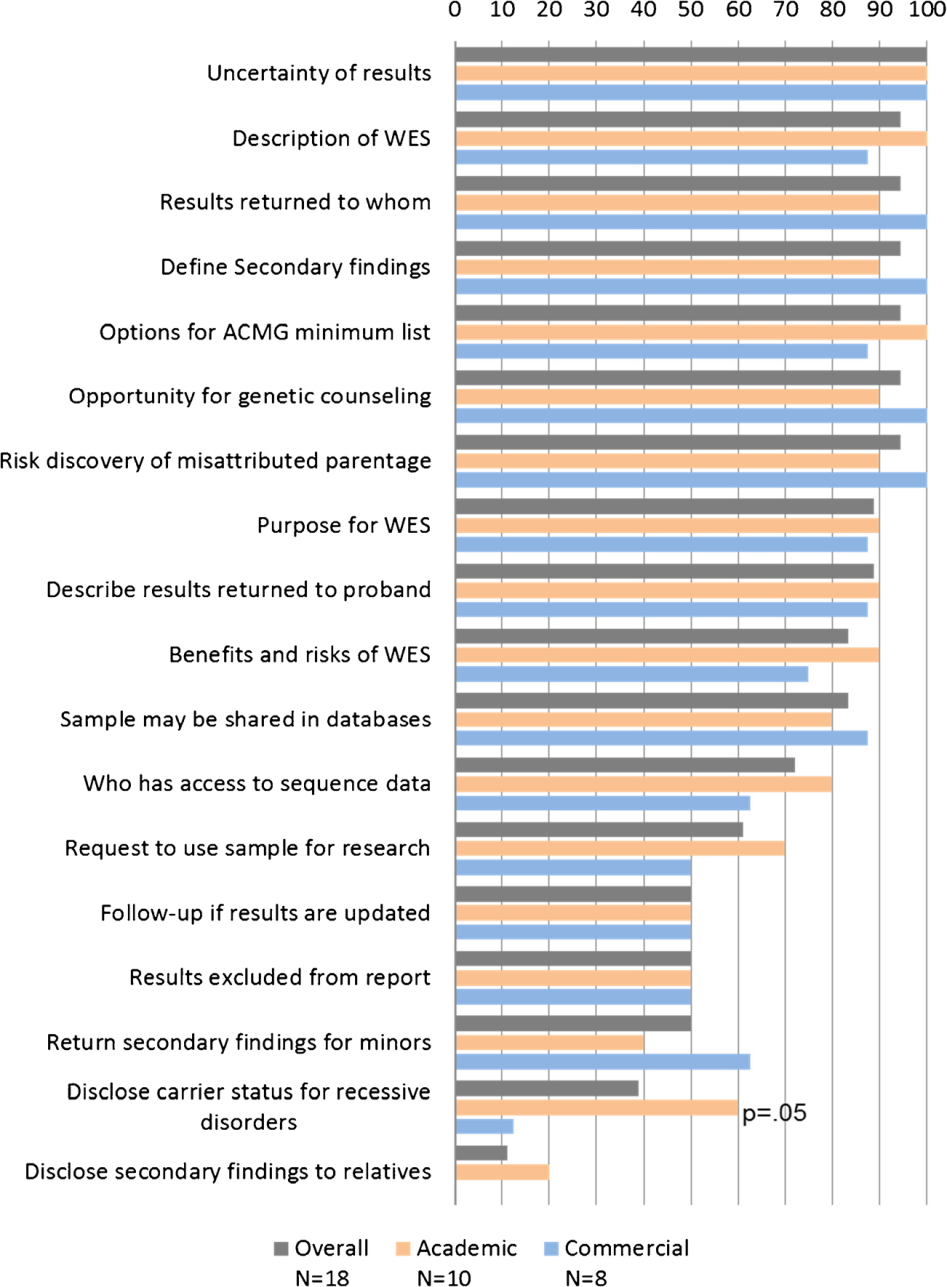
Frequency of individual bioethics commission and ACMG recommendations, overall and by laboratory type, *N* = 18

Recommendations of the ACMG and Bioethics Commission were present, to varying degrees, in all CFUs. For each CFU, the number of ACMG and Bioethics Commission recommendations ranged from 11 to 17 items (Mean = 13.44, Mode = 14) (Table 2). The frequency of each ACMG and Bioethics Commission recommendation ranged from 11.1% to 100% (Fig. 1).

**Table 2:**
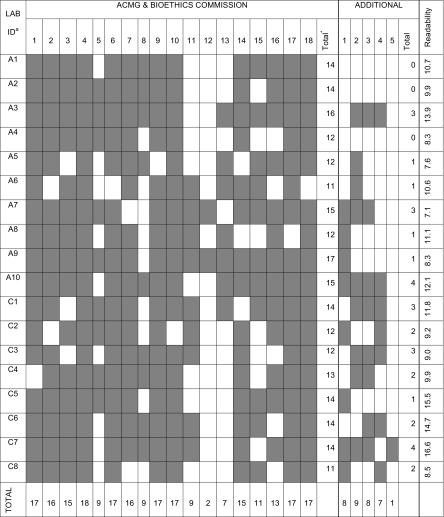
Matrix of inclusion of recommendations in each CFU per laboratory

*A = Academic, C = Commercial. Columns correlate with CFU numbers in Table 1. Shading indicates presence of recommendation

*Mean = 13.44, Mode = 14

All CFUs in the sample set disclosed potential uncertainty due to limitations of the test, such as DNA variants that are not detected with WES and/or limited ability to interpret the variants identified. Ninety-four percent of CFUs described WES, stated to whom results would be returned, defined SFs, explained options for receiving ACMG minimum list results, discussed opportunities for genetic counseling, and disclosed the risk of discovering misattributed parentage. The options for receiving ACMG minimum list results were: opt-out (33%), opt-in (28%), stratified choice (22%), and not offered (17%). Approximately 90% explained the purpose of WES and described the results to be included in the report. Eighty-three percent explained the benefits and risks of WES and disclosed that the de-identified data may be shared with national DNA databases. Seventy-two percent stated who may gain access to the sequence data. Approximately 60% of CFUs included a request to use the sample for research purposes. One-half discussed the possibility for follow-up communication if a new interpretation of results is learned, described results to be excluded from the report, or explained the approach to returning SFs for minors. Nearly 40% offered search and disclosure of results for carrier status for recessive disorders. Only 11% of CFUs included the recommendation that relevant SFs should be disclosed to relatives.

Among CFUs from academic laboratories, the inclusion of each ACMG and Bioethics Commission recommendation ranged from 20% to 100%. Academic laboratories more frequently described WES, provided opt-in or stratified choice options for seeking and reporting the ACMG minimum list, explained benefits and risks of WES, disclosed who would gain access to the sequence data, requested to use the remaining sample for research purposes, and explained that relevant SFs should be disclosed to relatives. Academic laboratory CFUs were significantly more likely than commercial laboratories to address the recommendation to disclose carrier status for recessive disorders (60% vs 12.5%, *p* = 0.05).

Among commercial laboratories, the inclusion of ACMG and Bioethics Commission recommendations ranged from 0% to 100%. CFUs from commercial laboratories more frequently provided opt-out or no offer for seeking and reporting the ACMG minimum list, defined SFs, described opportunities for genetic counseling, disclosed to whom results would be returned, explained the risk of discovering misattributed parentage, disclosed that de-identified data may be shared in DNA databases, and explained the policy for returning SFs to minors.

**Fig. 2:**
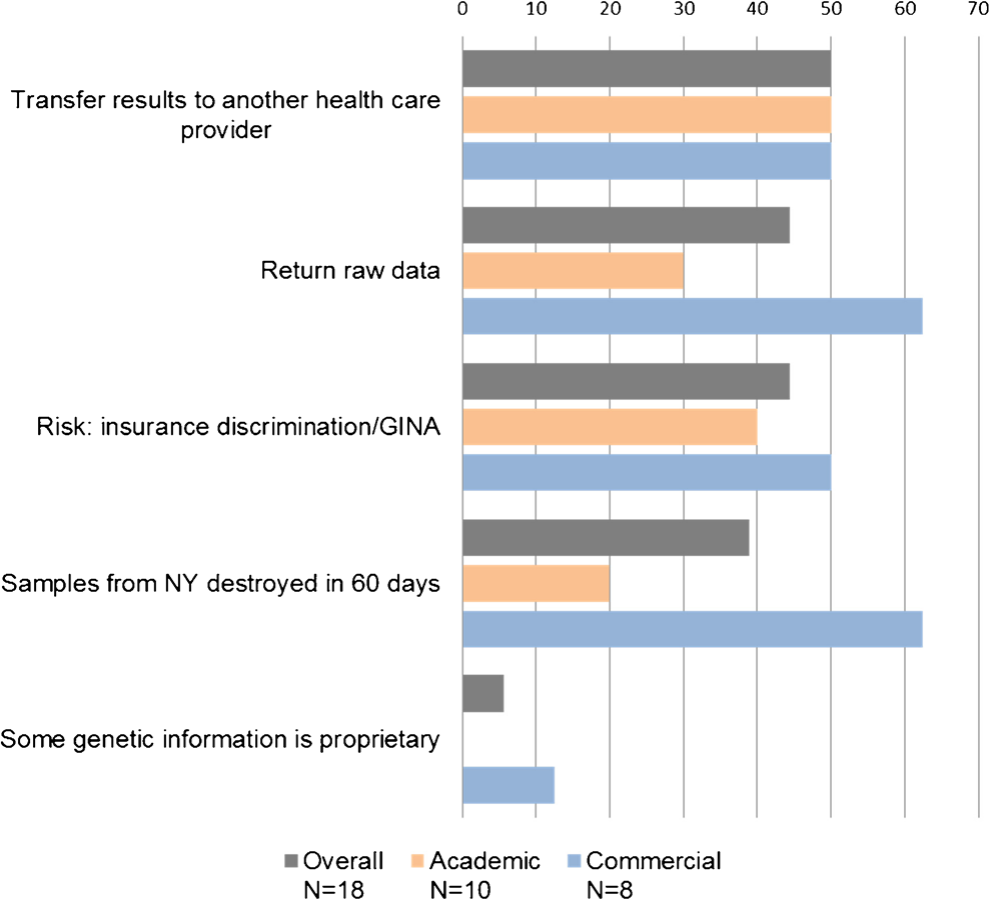
Frequency of other recommendations for informed consent, overall and by laboratory type, *N* = 18

We examined five additional recommendations for informed consent. The frequency of inclusion of these recommendations ranged from 0 to 4 items. One-half included a provision to transfer the WES results to another health care provider in addition to the ordering clinician (Fig. 2). Forty-four percent provided for return of the raw data file to the clinician and explained GINA. Although none of the laboratories in the study sample was located in New York, the state statute requiring that samples be destroyed within 60 days of testing was acknowledged in 39% of CFUs. One CFU (6%) informed patients that there may be proprietary data that could not be included in the analysis. Differences between laboratory types in inclusion of the five additional recommendations for informed consent were examined. Commercial laboratories more frequently offered the return of the raw data file, described GINA, explained the New York state statute, and discussed the possibility of proprietary genetic information.

The Flesch-Kincaid Grade Level scores of the CFUs ranged from 7.1 to 16.6 with a mean of 10.8. The grade level score for academic laboratory CFUs ranged from 7.1 to 13.9 with a mean of 10.0. The grade level score for commercial laboratory CFUs ranged from 9.0 to 16.6 with a mean of 11.9 (Table 2).

## Discussion

A prior study by Jamal et al. (2013) developed “core elements” of informed consent content based on a review of the literature, their own experience, and an iterative process with researchers, ethicists, and institutional review boards. These core elements were used to evaluate consent forms from six WES providers, and a wide variety of consent practices was found (Jamal et al. 2013). In another previous study, informed consent forms were reviewed to create a list of common elements that was then compared with a systematic review of the literature to identify the presence of each item within every document (Ayuso et al. 2013). This review found some general agreement on the content of consent forms for WES, but also a need to define policies and guidelines for the consent process (Ayuso et al. 2013). Unlike the earlier studies, our descriptive analysis uses the pre-defined guidelines of the ACMG and Bioethics Commission as benchmarks and compares the content used in practice by 18 laboratories against those benchmarks.

We found considerable variation in the use of consent features recommended by the ACMG and Bioethics Commission by 18 laboratories performing diagnostic WES. Our results indicate most of the 18 laboratories evaluated omit at least one of the recommendations of the ACMG and Bioethics Commission for informed consent of clinical WES. In particular, we found recommendations to follow-up if results are updated, explain results to be excluded from the report, return SFs for minors, disclose carrier status for recessive disorders, and disclose SFs to relatives were underrepresented in the CFUs that we evaluated.

We found that, on average, the CFUs were above the recommended 8th grade reading level, however, academic laboratories presented WES information in a manner more understandable to a patient with middle school literacy more often than commercial laboratories. The development of generic language could improve the readability of WES consent documents and help patients better understand the choices and consequences of WES.

We found that nearly all laboratories in the sample are integrating ACMG and Bioethics Commission guidance to describe how and why WES is conducted, define SFs and options for receiving these results, describe what results would be reported, and recommend genetic counseling. These issues are central to allowing patients to make an educated choice to have the clinical WES test and could be used as the basis for development of common language for consent (Berg et al. 2015; Gutmann 2013).

Policy and ethics professionals are involved in ongoing evaluation of specific criteria that can be used to determine when it is ethically permissible or obligatory for clinicians to disclose (or not disclose) SFs to patients (Green et al. 2013; Bioethics Commission 2013). Our results showed differences in the options laboratories provide to patients for the receipt of the ACMG minimum list of SFs, as well as opportunity for expanded SFs such as carrier status, reflecting this unsettled debate. Academic laboratories were more likely to provide patients with opt-in or stratified consent options to receive the minimum list or expanded SF results, including carrier status. This may be an indication that academic laboratory consent documents are designed to offer more flexibility and control to patients despite a potential increase in operating cost.

We found that one-half or more of CFUs did not discuss the return of SFs for minors or plainly recommend that patients should disclose relevant SFs to relatives. Laboratories that address the dilemmas of how to manage SFs for minors and/or the patient’s relatives demonstrate an approach to informed consent that is not yet well-established, perhaps because the guidance from several professional organizations provides conflicting advice (Ross et al. 2013). For example, the ACMG recommends avoiding arbitrary age limits in the reporting of SFs since these results may have implications for others in the family (Green et al. 2013). The American Society of Human Genetics acknowledges that physicians have a privilege, but not an obligation, to warn relatives of possible genetic risks in cases where the patient fails to voluntarily disclose to relatives (Wolf 2015). Conversely, the Institute of Medicine advises that genetic risk information should be withheld so as to avoid family disruption (Buchbinder and Timmermans 2011).

The understanding of results from WES will change as genomic technologies continue to develop (Bioethics Commission 2012). Indeed, disclosure of the uncertainty of results for WES was the only recommendation that was included in every CFU, regardless of laboratory type. A related issue is the need to follow up with patients as new knowledge becomes available. However, an explanation that the laboratory may re-analyze the data after a certain period of time and re-contact the ordering clinician if a new interpretation of results is learned was included in just one-half of CFUs from both laboratory types. This discrepancy deserves further attention in future professional guidance. As WES becomes more widely used, laboratories will need to understand their responsibility to apprise patients of new information, and patients will need to understand their responsibility to seek updated results (Ayuso et al. 2013).

One well-established component of informed consent is the disclosure of benefits and risks (Bioethics Commission 2012; OHRP 1993). However, not all CFUs included a specific section to explain benefits and risks of WES. In some instances, the risk of discovering misattributed parentage was embedded with the results to be returned to the patient. Some CFUs omitted an explanation of possible psychological risks of WES such as feeling frustrated, angry, disappointed, or depressed related to the results. The use of an explicit section with standard language describing risks of WES is an aspect of informed consent that could be improved.

According to the OHRP (2009), consent processes should reflect the protections provided by GINA. Our study found that 56% of laboratories did not include information about GINA. This omission is a potentially serious oversight as it could have financial and/or health care repercussions for those who receive SFs.

Patients undergoing WES may be experiencing a diagnostic odyssey, attempting to establish a definite diagnosis for a rare disease or complex condition. At the same time, the interpretation of clinical WES results is evolving. Consequently, patients may want to explore other clinical opinions of the information (analyzed data interpreted by experts) and raw data (unanalyzed sequence data) received from the test (Bioethics Commission 2012). The Bioethics Commission (2012) recommends patients be informed of what data and information may be returned to the individual; the ACMG elected not to consider the question of returning raw data (Green et al. 2013). In our study, commercial laboratories were more likely to permit the return of raw data, and sometimes specifically identified the data file format as FASTQ, Binary Alignment Map (BAM), and/or Variant Call Format (VCF). Professional standards to offer the option for transfer of information and return of raw data to clinicians, including file format, could allow patients to receive maximum benefit from WES.

Another variation among WES consent forms was related to the handling of the New York state law requiring that biological samples be destroyed within 60 days of testing (New York Department of Health 2011). While none of the study laboratories were located in New York, we found that nearly two-thirds of commercial and one-fifth of academic laboratories cited this law. This discrepancy may reflect that commercial laboratories often have a broader service catchment area than academic laboratories. If more states enact state-specific legislation for genetic testing, the informed consent process will need to be attentive and responsive to changing laws.

A second legal matter pertains to protections for intellectual property and patent laws. Myriad Genetics, Inc. sought to enforce patent rights to the *BRCA1* and *BRCA2* genes, bringing litigation against laboratories performing sequencing of these genes (Sherkow and Greely 2015). The US Supreme Court’s recent decision in *Association for Molecular Pathology v. Myriad Genetics, Inc.* established that methods of conducting genetic risk-assessment are not eligible for patent claims (Sherkow and Greely 2015). However, patents on the use of specific genes for gene therapy continue to be possible and patent protection for them could come to be significant (Sherkow and Greely 2015). Only one CFU in our sample, a commercial laboratory, included language disclosing the possibility of proprietary data which could not be used in the analysis of results. This was a notable exception among the sample of CFUs. It is unknown whether the resolution of the Myriad case may negate the future need to include this type of statement.

In our study, we used the ACMG and Bioethics Commission recommendations as benchmarks but did not have visibility into the extent to which any of the laboratories reviewed these recommendations or used them to shape the language of their consent forms. The gaps between the consent forms and the recommendations could reflect lack of awareness, intentional departure from the recommendations or recognition that a lab needs more time to align their practices with the recommendations.

### Practice Implications

The informed consent process is the principal opportunity for communication between clinicians and patients (Bioethics Commission 2013). Clinicians need to provide patients with sufficient information to make educated decisions about the treatment they receive (Bester et al. 2016; Crawford et al. 2013; Simon et al. 2011). Our study demonstrates that patients receive different information for the consent of clinical WES depending on which laboratory conducts the procedure. Consensus is lacking on what information will be sought, how to respect individual patient preferences, and which SF results will be returned (Green et al. 2013; Gutmann 2013; Klitzman et al. 2013). In order to improve the quality of communication between clinicians and patients, existing guidelines, such as those provided by the ACMG and Bioethics Commission, should be utilized more widely in practice. Our work provides a consolidated checklist that laboratories offering WES can utilize to align their consent forms with these benchmarks.

### Study Limitations

The study had a limited scope, including only informed consent documents for clinical WES that were available for public download. Additional laboratories were identified as providing clinical WES that did not post their consent forms online. The scope of inclusion of ACMG and Bioethics Commission recommendations may be different among laboratories that limit access to their informed consent forms. The small sample size limited the statistical power to detect meaningful differences.

The study identified laboratories that conduct clinical WES by searching online genetic test registries. Registration in databases such as NCBI’s Gene Test Registry is voluntary, thus the sampling of laboratories reflects those that choose to submit information to such databases. It is unknown how many laboratories actually conduct the clinical WES procedure. The study sample may be biased toward larger laboratories and exclude small or private organizations.

Another limitation is that the content analysis was conducted by a single evaluator. While steps were taken to improve the validity and reliability of the ratings by repeating the assessment of forms, it is possible that another analyst could interpret consent language differently.

### Research Recommendations

Future studies that include consent forms from laboratories that limit online access to their forms and/or are not listed in genetic test registries are needed to better evaluate the spectrum of informed consent documents. Studies involving patients (or prospective patients) are needed to compare different consent content and patient choices to understand the associated benefits, risks, and costs. Usability studies would provide rich information about comprehension.

## Conclusion

We observed considerable variability in the content of informed consent documents among the sample of 18 laboratories. This analysis will be useful to laboratories that provide clinical WES in designing informed consent forms in alignment with recommendations from the ACMG and Bioethics Commission. The development of a more standardized informed consent process could improve communication between clinicians and patients and increase understanding of WES.
